# Effects of lotus seedpod oligomeric procyanidins on the inhibition of AGEs formation and sensory quality of tough biscuits

**DOI:** 10.3389/fnut.2022.1031550

**Published:** 2022-10-06

**Authors:** Ziting Chen, Jiangying Tan, Jiabin Qin, Nianjie Feng, Qianting Liu, Chan Zhang, Qian Wu

**Affiliations:** ^1^Key Laboratory of Fermentation Engineering (Ministry of Education), National “111” Center for Cellular Regulation and Molecular Pharmaceutics, Cooperative Innovation Center of Industrial Fermentation (Ministry of Education & Hubei Province), Hubei Key Laboratory of Industrial Microbiology, Hubei University of Technology, Wuhan, China; ^2^Beijing Laboratory of Food Quality and Safety, School of Food and Chemical Engineering, Beijing Technology and Business University, Beijing, China

**Keywords:** lotus seedpod oligomeric procyanidins, tough biscuit, advanced glycation end products, sensory quality, inhibition

## Abstract

The advanced glycation end products (AGEs) are formed in baked products through the Maillard reaction (MR), which are thought to be a contributing factor to chronic diseases such as heart diseases and diabetes. Lotus seedpod oligomeric procyanidins (LSOPC) are natural antioxidants that have been added to tough biscuit to create functional foods that may lower the risk of chronic diseases. The effect of LSOPC on AGEs formation and the sensory quality of tough biscuit were examined in this study. With the addition of LSOPC, the AGEs scavenging rate and antioxidant capacity of LSOPC-added tough biscuits were dramatically improved. The chromatic aberration (ΔE) value of tough biscuits containing LSOPC increased significantly. Higher addition of LSOPC, on the other hand, could effectively substantially reduced the moisture content, water activity, and pH of LSOPC toughen biscuits. These findings imply that using LSOPC as additive not only lowers the generation of AGEs, but also improves sensory quality of tough biscuit.

## Introduction

MR is a complex crosslink of continuous and parallel reactions triggered by the condensation of a protein's amino residue and a sugar's carbonyl contraction, resulting in the formation of a multitude of compounds known as MR products (1). The MR not only plays a pivotal role in the textural, flavor, unique color, and nutritional properties in the thermal processing of foods (2), but is also accompanied by some potential chemical hazards such as AGEs (3).

Recently, AGEs have been researched to be involved in the pathogenesis of diabetic complications, such as retinopathy, nephropathy, and other diseases including atherosclerosis and other phenotypes related to aging (4). When accumulating in the human body, AGEs with functional proteins will form complex cross-links that change the proteins' structural makeup and shift their biochemical functions, which can cause diabetic complications and certain other health problems (5). In the process of glycosylation, an abundance of reactive oxygen species are produced, increasing the oxidative stress in the body. In cell culture studies, AGEs were discovered to induce cellular oxidative stress and cell activation. In some epidemiological studies, excessive intake of AGEs in the diet was also believed to increase inflammation and oxidative stress (6, 7).

Baked products, especially biscuits and bread, have been used as replicable food models. During the brewing process of baking, the protein and sugar in raw materials will undergo MR, forming a unique color and flavor (8). Meanwhile, some potentially harmful substances, including a whole load of 5-hydroxymethylfurfural (5-HMF) and AGEs will also be generated and cause the food safety hazards. The MGO content was 727–1,397 μg/100 g and the GO content was 338–1,936 μg/100 g in salty biscuits (9). In view of previous research reports, many phenolic compounds have been studied to have antiglycation effects under simulated physiological conditions (10). Therefore, it is necessary to inhibit the AGEs formation in biscuits. In general, the phenolic compounds' antiglycative activity is attributed to the capture of dicarbonyl compounds and antioxidant activity by scavenging free radicals and metal ion chelation (11, 12). LSOPC is a sort of procyanidin extracted from the mature receptacle of lotus house and prosses multiple biological activities, including antioxidant, anti-age, anti-cancer, and anti-glycated effects (13–17). Due to their powerful antioxidant activity and a wide range of meaningful biological functions, they have attracted widespread attention.

The effect of LSOPC in preventing AGEs in simulated food systems had been the subject of an increasing number of studies in recent years. In lactose-lysine simulated system, in different pH, temperature, metal ions environment and procyanidin concentration, lotus procyanidins can inhibit the AGEs formation effectively (18). Additionally, other model systems were selected consisting of lysine (as a very reactive amino acid) and maltose/glucose (as a reducing sugar) to monitor the CML and AGEs formation, and observe the inhibition of LSOPC on AGEs and CML formation (19). However, for tough biscuits, there had been little discussion could comprehensively evaluate the inhibitory relationship between LSOPC and AGEs and the effect of LSOPC on sensory quality.

**Scheme 1:**
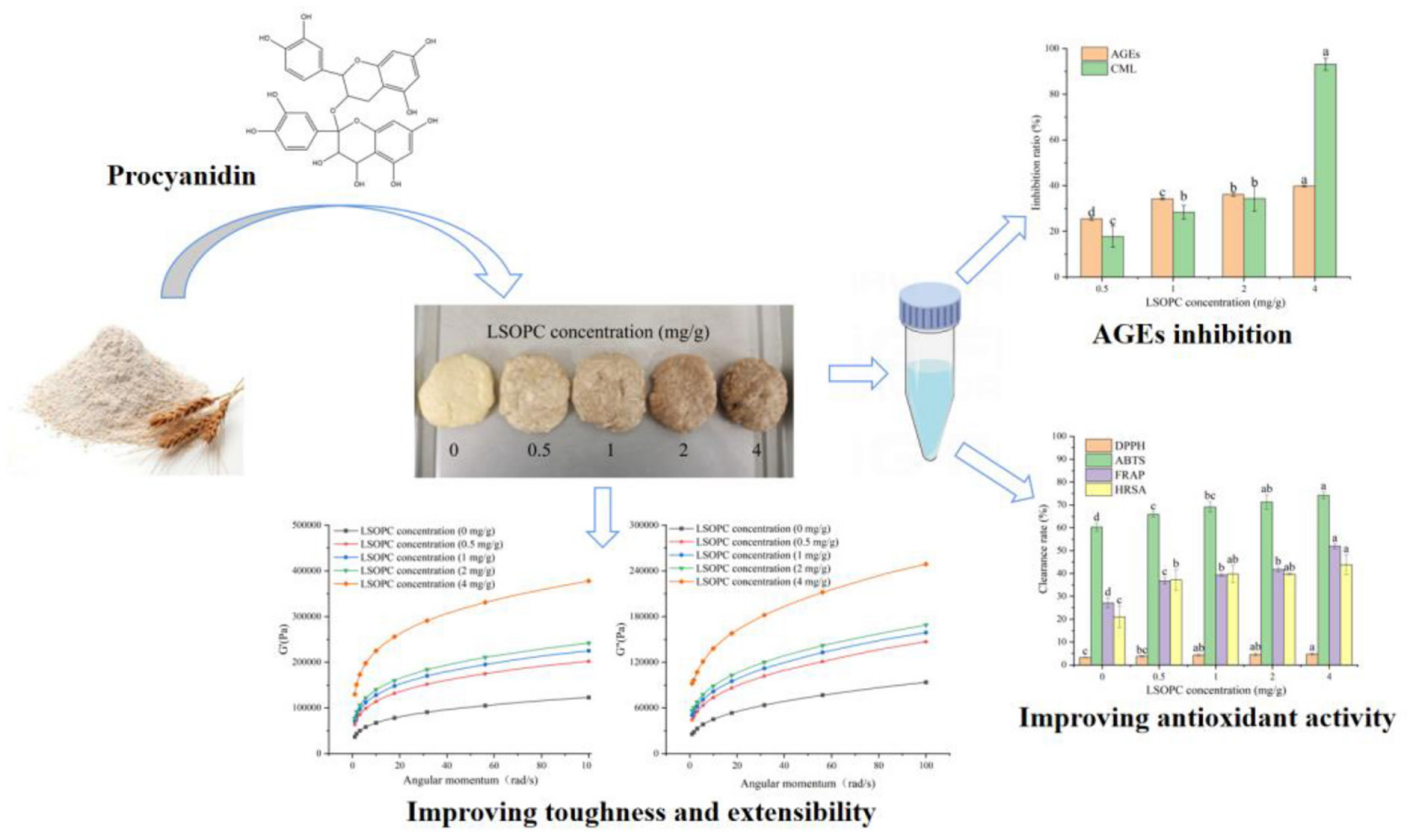
Effect of LSOPC on tough biscuits.

In this research, the inhibitory effect of LSOPC on tough biscuits AGEs formation was investigated by detecting the inhibitory rate of LSOPC on fluorescent AGEs and CML formation. In addition, this research also investigated the effect of LSOPC on the sensory quality of tough biscuits by examining pH, moisture, chroma, electronic nose, and rheology Scheme 1). Taking into consideration all the facts stated above, the objective of this study provided the theoretical base for LSOPC as a sort of functional food additives and improved reference for the effects of high value application of LSOPC.

## Materials and methods

### Materials

Low-gluten wheat flour and sugar were purchased from Angel Yeast Co., Ltd (Yichang, China). Milk was obtained from Weidendorf (Shanghai, China). Eggs were bought from a local supermarket. Sodium bicarbonate food grade, ammonium bicarboante (food grade), Tween-20, sodium dodecyl sulfate (SDS), β-mercaptoethanol, tris (hydroxymethyl) aminomethane-HCl (Tris–HCl), hydrogen peroxide (H_2_O_2_), folin-phenol,2,2-diphenyl-1-picrylhydrazyl (DPPH), potassium persulfate (K_2_S_2_O_8_), alicylic acid, ferrous sulfate and 2,2'-azinobis-(3-ethyl-benzothiazoline-6-sulfonic) acid (ABTS) were obtained from Sinopharm Chemical Reagent Factory (Shanghai, China). Sodium acetate, 2,4,6-tripyridyl-s-triazine (TPTZ), ferric chloride and ethanol were purchased from Sigma-Aldrich (St Louis, MO). Standard N-ε-carboxymethyl lysine (CML) was purchased from Toronto Research Chemicals (Toronto, Canada). In addition, solvents and chemicals such as methanol (HPLC grade), chloroform (HPLC grade) and sodium borohydride solution (pH 13–14) were supplied by Fisher Scientific (Fairlawn, NJ, USA). The other chemicals were of analytical grade.

### Prepare of biscuits

The model biscuits were produced as Gökmen et al. (20) description with modifications. The following ingredients were used to create the recipes: 12.3 grams of wheat flour, 2.5 grams of sucrose, 2.5 grams of deionized water, 2.5 grams of sunflower oil, 0.06 grams of sodium bicarbonate, 0.09 grams of ammonium bicarbonate, and 2.5 grams of salt (control dough). LSOPC was evenly mixed into soft flour at a series of gradient concentrations (0, 0.05, 0.1, 0.2, and 0.40 mg/g). After the ingredients had been well combined, the dough was rolled out to form disk that were 5 cm in diameter and 4 mm thick. Samples were then baked for 15 mins at 170°C. The biscuits were made in triplicate and baked in a natural convection oven (Memmert UNE 400, Germany). Ground biscuits (0.2 g) were added with water (10 mL) and ultrasonically shocked for 1 h prior to centrifugation at 25°C, 3,000 rpm for 5 mins, and collecting liquid for sample extraction.

### Determination of the fluorescent AGEs

The procedure was based on previous methods by Zhang et al. (21). with some modifications. Samples were gained from different concentration stages. They were then added in the 96-well microplate for quantitatively assessing the fluorescent AGEs' formation using a spectrophotometer (Shimadzu RF-5301) with wavelengths of 370 nm/440 nm for the excitation and emission, respectively. As a control, reaction solution devoid of LSOPC was utilized. The inhibition was calculated as:


$$\begin{array}{l} Inhibition\;\left(\%\right)=\frac{A_{control}-A_{sample}}{A_{control}}\times 100 \end{array}$$

A_control_ and A_sample_ were the sample's and the control's absorbance, respectively.

### N-ε-carboxymethyl lysine (CML) determination

Reducing reagent of 2 mL sodium borohydride (pH 13, prepared with 0.1 M NaOH) were joined to the dried sample (0.5 g) for 10 h at 4°C. The supernatant was gained by centrifugation and then passed through the preactivated solid-phase extraction PCX column (22). Prior to HPLC-MS2 analysis, the eluted substance was resuspended in 1 mL of 0.1% formic acid and filtered over a 0.22 μm organic membrane. There were three parallel experiments run.

With 0.2% formic acid (solvent A) and acetonitrile (solvent B) acting as the mobile phases, 15 mL of sample was then injected into an Eclipse Plus C18 column (2.1 × 50 mm, 5 μm, Agilent Technologies, Germany) at 30°C. To achieve satisfactory separation, the chromatographic settings were adjusted to a run length of 25 min and a flow rate of 0.2 mL/min. Following were the parameters of the gradient program: 0–0.5 min, 90% A; 0.5–4.0 min, 90–60% A; and 4.0–25.0 min, 60% A. The positive ion mode was used to operate the mass spectrometer with multiple reaction monitoring. The capillary voltage was held at 4 kV and the nitrogen temperature was maintained at 300°C. The fragments at m/z 84 and 130 were employed for the quantitative and qualitative analyses of CML, respectively. (Agilent Technologies, Germany).

### Determination of total phenol content (TPC)

The 0.2 mL standard solutions with concentrations of 0, 50, 100, 150, 250 and 500 mg/L were taken out and mixed with 12 mL deionized water. After 1 mL of folin-phenol reagent was added, it was mixed evenly for 30 s, then allowed to stand for 8 min, and finally 3 mL of 20% sodium carbonate solution was added to make up to 20 mL. After 2 h of equilibration at ambient temperature, the colorimetric method was performed at 765 nm (23).

### Analysis of LSOPC degradation

LSOPC's concentration at 546 nm was measured using a UV spectrometer (24). The standard curves were created using standard substances under identical circumstances. The deterioration was calculated as follows:


$$\begin{array}{l} \text{Degradation }\left(\text{\%}\right)=\frac{{\text{C}}_{\text{a}}-{\text{C}}_{\text{b}}}{{\text{C}}_{\text{b}}}\times 100 \end{array}$$

LSOPC concentrations with treatment and without treatment were C_a_ and C_b_, respectively.

### Analysis of the biscuits' functional properties

#### DPPH radical scavenging assay

According to Mensor et al. the radical scavenging activity was assessed using a DPPH test (25). Three copies of each test were run for each experiment. The antioxidant activity was calculated as follows:


$$\begin{array}{l} \text{DPPH radical scavenging activity }\left(\text{\%}\right)=1-\frac{\left(\text{A}-{\text{A}}_{\text{b}}\right)}{{\text{A}}_{0}}\times 100 \end{array}$$

Where A_0_ represented the absorbance of DPPH at 517 nm, A represented the absorbance of the sample and DPPH at 517 nm, and A_b_ represented the absorbance of the sample at 517 nm.

#### ABTS radical scavenging assay

As described by Sun et al. the ABTS^+^ radical scavenging ability was measured (26). Three parallel experiments were performed. The ABTS radical scavenging activity was calculated as follows:


$$\begin{array}{l} \text{ABTS radical scavenging activity }\left(\text{\%}\right)=\frac{A_{control}-A_{sample}}{A_{control}}\\ \;\times 100 \end{array}$$

Where A_control_ represented the absorbance of ABTS and A_sample_ represented the absorbance of the sample and ABTS^+^ at 734 nm.

#### FRAP assay

The FRAP activity was measured as Balkan et al. described (27). Three parallel experiments were performed. The Ferric reducing antioxidant power was calculated as followed:


$$\begin{array}{l} \text{Ferric reducing antioxidant power }\left(\text{\%}\right)=\frac{A_{sample}-A_{control}}{A_{black}}\\ \;\times 100 \end{array}$$

Where A_control_ was the absorbance of FRAP at 593 nm, A_sample_ was the absorbance of the sample and FRAP at 593 nm, A_black_ was the absorbance of the sample at 593 nm.

#### HRSA

The HRSA was measured as Meng et al. discribed (28). The hydroxyl radical scavenging rate was expressed as % using the following formula:


$$\begin{array}{l} \text{Hydroxyl radical scavenging rate }\left(\text{\%}\right)=1-\frac{{\text{A}}_{1}-{\text{A}}_{2}}{{\text{A}}_{0}}\times 100 \end{array}$$

Where A_0_ was the absorbance at 510 nm without a sample, A_1_ was the absorbance of the sample at 510 nm, and A_2_ was the absorbance of the sample without H_2_O_2_ at 510 nm.

### Determination of water

#### Determination of Aw

Water activity was determined at 25°C using the AquaLAB CX-2 (Decagon Devices Inc., Pullman, WA).

#### Determination of moisture content

Samples was determined by Moisture meter (200 mg, 105°C).

#### Nuclear magnetic resonance

The biscuit was removed and transferred to the NMR tube, and the water distribution was measured by nuclear magnetic resonance imaging analyzer. The test conditions were as follows: the number of sampling points was set to 1,024, the number of repeated scans was set to 8, and the relaxation attenuation time was set to 2,000 ms (29). The relaxation time T2 was determined by CPMG pulse sequence.

### Determination of pH

Prior to measurement, ground biscuits (0.1 g) were mixed with 10 mL of water and ultrasound for 3 mins(37°C). The mixture was centrifuged at 25°C, 3,000 rpm for separating liquid and solid phases. And after separating the supernatant from the precipitate, the pH of the supernatant was determined using a portable pH meter.

### Determination of color

Cookie chroma was measured according to the method of Siti Rashima (30). The color of biscuits was measured by a Konika Minolta reflectance spectrophotometer CM-3500d (Konica Minolta Sensing INC, Osaka, Japan) and the results were expressed using the CIE Lab color system. The three independent measurements of parameters a^*^ (redness), b^*^ (yellowing), and L^*^ (lightness) were conducted at different locations of the biscuit surface. The value E was calculated according to the equation:


$$\begin{array}{l} △E=\sqrt{\left(△L^{*2}+△a^{*2}\;+{△b}^{*2}\;\right)} \end{array}$$

### Texture determination

The probe, which had a P/36R cylindrical design, had a compression speed of 5 mm/s, a pre- and post-test speed of 5 mm/s, as well as a sensing force of 5 g. Each sample was measured three times, with a 50% target mode strain (31). The disparity between the peak load reported by the texture analyzer and the ultimate load was used to assess the homogeneity of several doughs.

### Electronic-nose data acquisition

The electronic nose type was a portable PEN3 (Airsense, German) electronic nose that contained 10 metal oxide gas detectors. The 10 gas sensors were fitted onto a printed circuit board (32). Gas sensor metering, and each detection room have been separated, helping to avoid cross-influence of gas flow. Each sample was placed in a 30 mL airtight vial and sealed with a sealant membrane. The flasks were balanced for 30 mins at 65°C using a magnetic agitator. The gases in the sample head space were pumped into a gas sensor chamber by a sampling pump at a flow velocity of 400 mL min^−1^ for 100 s. Electronic-nose real-time responses to the sample were recorded.

### Determination of the rheological propriety

A DHR-3 rotary rheometer (TA Instruments Inc., USA) was used to determine the dynamic rheologic properties of the samples. The test condition included the plate diameter of 40 mm, the gap of 1 mm, the temperature at 25°C, the scanning strain set to 1%, and the frequency at 0.1–10 Hz. At last, the spectra of the storage module (G') and the loss module (G”) were obtained.

### Statistical analyses and graph drawing

All of the tests and analyses were conducted in triplicate. The data were analyzed by using SPSS v21.0 (expressed as mean ± SD), the differences were considered statistically significant at *p* < *0.05*. Graphs were generated using Origin Pro 8.0.

## Results and discussion

### The effect of LSOPC on fluorescent AGEs and CML formation

Generally speaking, AGEs can be classified into two groups in terms of chemical structure and property (33). One type is fluorescent cross-linking AGEs, including glyoxal lysine dimer and cross-linking element, and the other category is non-fluorescent non-cross-linking architectures, such as CML and carboxyethyl lysine (CEL) (34).

**Figure 1:**
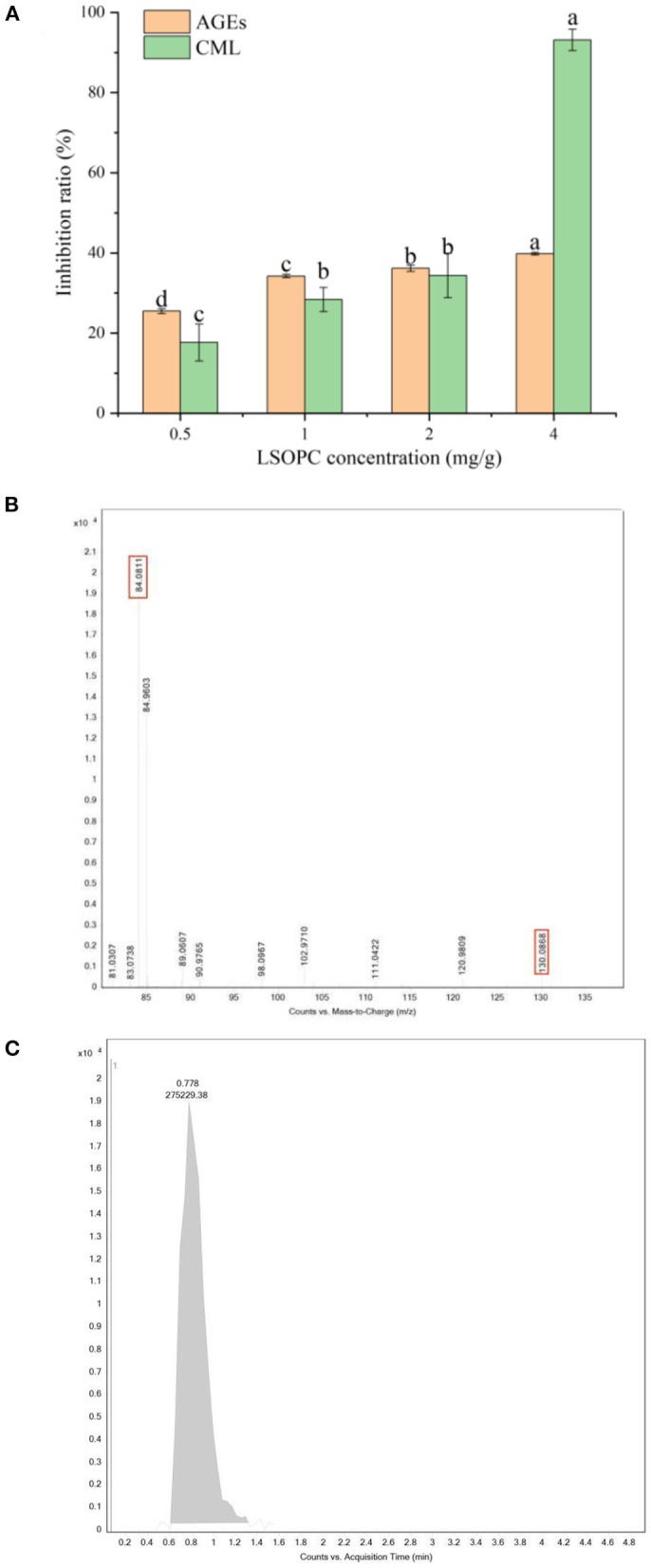
Effect of different concentrations of LSOPC on inhibition rate of AGEs and CML in biscuits **(A)**. CML: ion spectra of the standard substance **(B)**. CML: peak area of the standard substance **(C)**. Significant differences (*p* < 0.05) of data values were indicated by different letters.

As shown in Figure 1A, the formation of fluorescence AGEs was significantly inhibited by LSOPC. When the dough was formulated with 4 mg LSOPC/g biscuits, the formation of fluorescent AGEs was significantly reduced to 39.79 ± 0.34%. The inhibitory capability against the formation of fluorescent AGEs was positively connected with the concentrations of LSOPC, indicating that LSOPC was found to be able to prevent the formation of fluorescence AGEs during biscuits baked. These outcomes were in line with that published by Culetu er al. (35) who demonstrated that the addition of polyphenol-rich fractions from decaffeinated tea dust in the formulation of bread allowed for a reduction in the formation of fluorescent AGEs.

Figure 1A showed the changes of CML contents in biscuit after the addition of LSOPC (0–0.4 mg/g). The initial contents of CML in biscuit were 0.0403 ± 0.0007 ug/mL. For subsequent biscuit with LSOPC addition, the content of CML had a significant change, which indicated that CML content significantly decreased from 0.0403 ± 0.0007 ug/mL (0 LSOPC mg/g biscuits) to 0.017 ± 0.0005 ug/mL (4 LSOPC mg/g biscuits). Furthermore, the CML inhibition rate also increased over this period, rising from 17.67 ± 4.62% to 93.14 ± 2.66%. In this regard, our findings were consistent with Peng et al. (36) who claimed that bread fortification with grape seed extract inhibited CML formation.

In conclusion, the inhibition of fluorescent AGEs and CML were positively correlated with the concentration of LSOPC (*p* < 0.05), indicating that LSOPC had a significant inhibitory effect on AGEs (*p* < 0.05).

### The effect of LSOPC on total phenol content (TPC)

**Figure 2:**
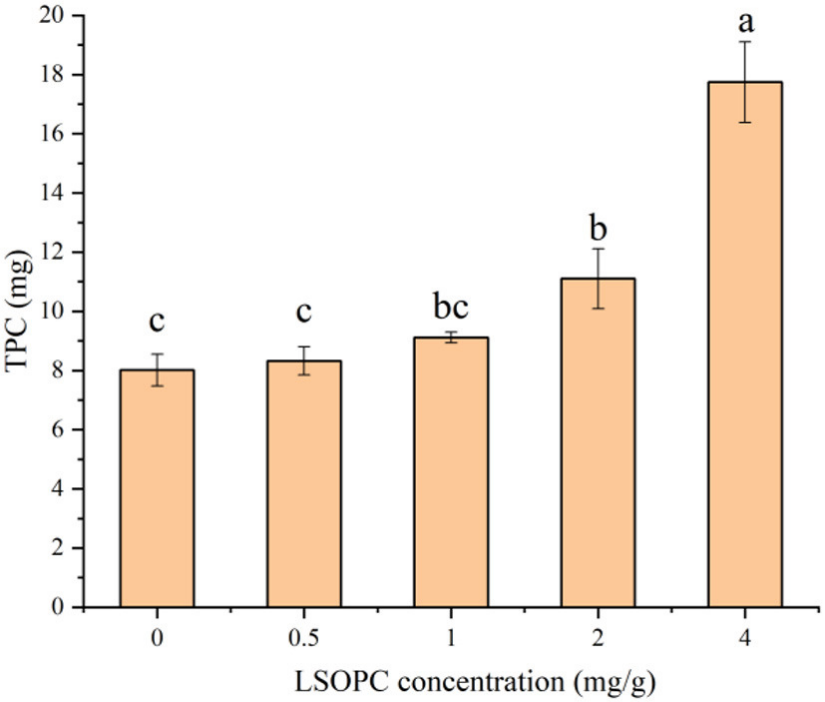
Effect of different concentrations of LSOPC on total phenol content in biscuits. Significant differences (*p* < 0.05) of data values were indicated by different letters.

The TPC of biscuit with LSOPC addition was shown in Figure 2. The higher LSOPC concentration contained significantly higher (*p* < 0.05) total phenolics (17.74 mg/g) in biscuits. Highest LSOPC concentration biscuit possessed the highest level of polyphenol 17.74 mg/g (4 LSOPC mg/g biscuits), followed by 11.10 mg/g (2 LSOPC mg/g biscuits), 9.11 mg/g (1 LSOPC mg/g biscuits) and 8.32 mg/g (0.5 LSOPC mg/g biscuits) with substantial variations between each other (*p* < 0.05). The TPC intensity of the samples made by the four concentrations of LSOPC biscuits in this investigation demonstrated a favorable correlation with concentration in the biscuit-making process, indicating that although the vast majority of LSOPC was consumed, there was some residual.

### LSOPC degradation in biscuit

**Figure 3:**
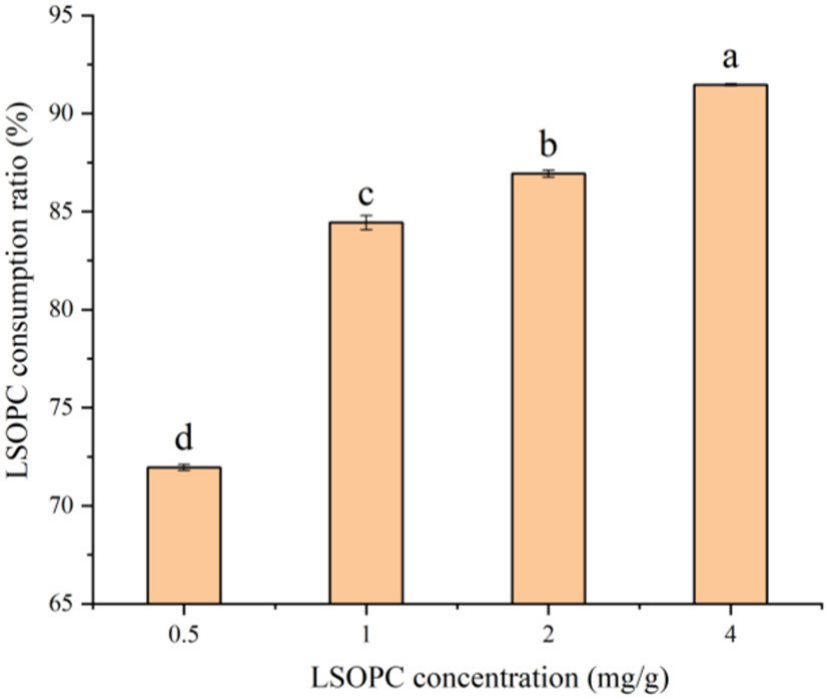
Effect of different concentrations of LSOPC on its consumption ratio in biscuits. Significant differences (*p* < 0.05) of data values were indicated by different letters.

LSOPC degradation in biscuit showed positive trend with increasing LSOPC concentration (*p*<*0.05*), which was illustrated in Figure 3. Combining Figure 1, it can be found that the more LSOPC was added, the higher the LSOPC consumption rate, thereby providing a valuable reference on the stronger inhibition of AGEs in biscuit system. LSOPC significantly restrain the oxidation reaction in the MR as more of LSOPC interacted with the MR. Therefore, LSOPC significantly suppressed the generation of AGEs and CML, which were the products of the MR. This conclusion was consistent with the above-mentioned that the significant rise in the inhibition of AGEs and CML was associated to the increase in LSOPC concentration.

### Effect of LSOPC on the antioxidant activity of biscuit

**Figure 4:**
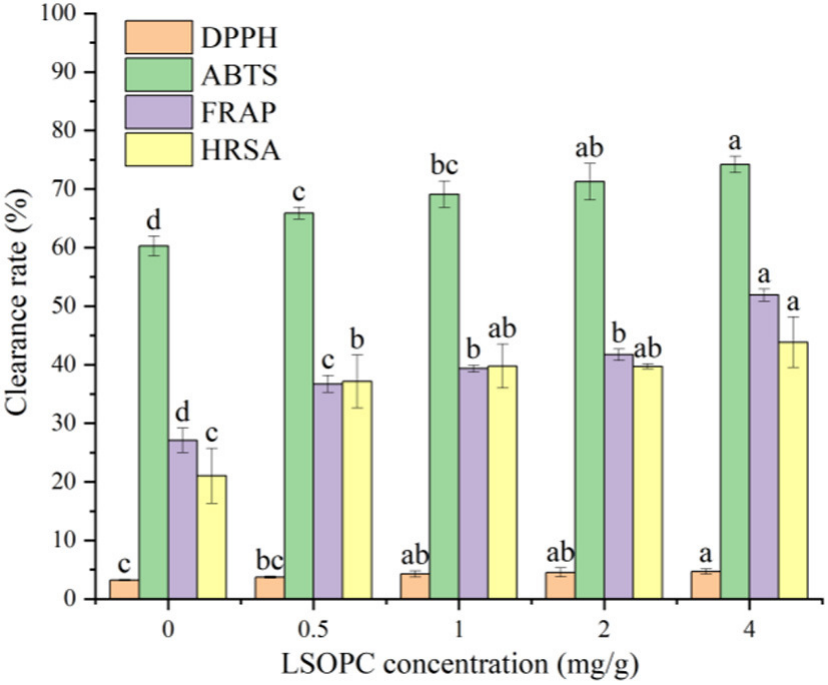
Effect of LSOPC on DPPH, ABTs, HRSA, FRAP scavenging activity. Significant differences *(p* < 0.05*)* of data values were indicated by different letters.

Evidence from numerous *in vitro* and *in vivo* studies identified that AGEs formation had a closely connection with carbonyl stress and oxidative stress (37). To investigate the impact of LSOPC on antioxidant capacity of biscuit, four antioxidant indexes were selected, including DPPH, ABTS, FRAP and HRSA. Results in Figure 4 showed that the incorporation of LSOPC in biscuit led to a higher antioxidant capacity compared to the control (0 LSOPC mg/g biscuits). Significant differences in DPPH, ABTS, FRAP and HRSA were observed between each other (*p* < 0.05). The maximum DPPH radical scavenging activity, FRAP and HRSA were found in the biscuit containing 4 mg/g LSOPC, up to 4.70 ± 0.46, 51.88 ± 1.03, and 43.84 ± 4.32%, respectively. Meanwhile, the addition of LSOPC had the most remarkable impact on the ABTS of biscuit, it increased from 60.26 ± 1.69 to 74.20 ± 1.43% after adding LSOPC. Combining the results of the four different antioxidant tests with the TPC content, it can be revealed that the high LSOPC-added tough biscuits had the greatest TPC level and the best antioxidant characteristics. Thus, there was a significant correlation between the antioxidant capacity and TPC indicating that phenolic chemicals are the primary contributors of this action (38).

The supplementation of functional ingredients or foods has recently gained a lot of attention. Zhu et al. improved the antioxidant qualities of Chinese steam bread by incorporating black tea (39). Likewise, the antioxidant activity of the whole-wheat flour was successfully boosted by mixing green tea powder into it (40). The aforementioned findings demonstrated that LSOPC can enhance the functional activity of tough biscuits by acting as a functional factor in addition to increasing the antioxidant and decreasing AGEs content. Strong antioxidant substances that were exogenously administered could successfully inhibit AGEs formation (41). Therefore, the addition of LSOPC might prevent the formation of AGEs by the antioxidation.

### Water and low-field NMR properties

**Figure 5:**
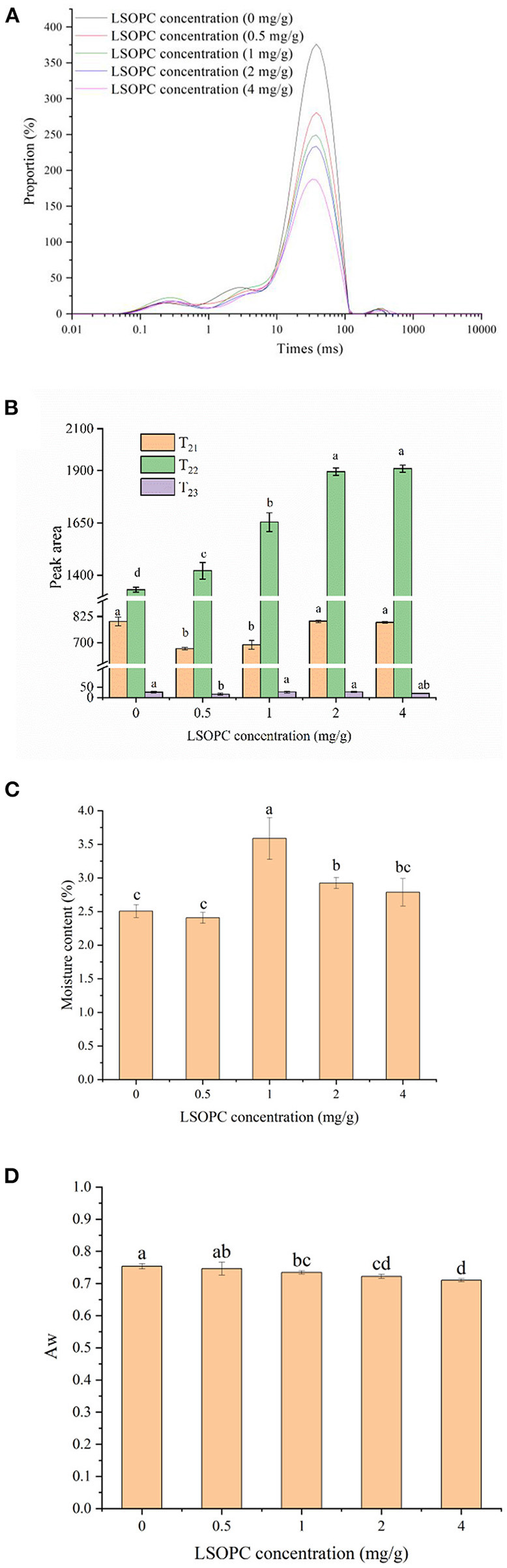
Effect of LSOPC on relaxation time **(A)**. Effect of LSOPC on peak area **(B)**. Effect of LSOPC on moisture content **(C)**. Effect of LSOPC on Aw **(D)**. Significant differences (*p* < 0.05) of data values were indicated by different letters.

The NMR signal decline could typically be described by a dispersed exponential with one to three distinct peaks. T_2_ relaxation periods measured on the LSOPC biscuit models were distributed in Figure 5A. At all concentrations, three different water populations with centers at roughly 0.1–10 ms (T_21_), 10–100 ms (T_22_), 100–1,000 ms (T_23_) were detected. The components and population of existed in each water fraction were denoted by the water that found in macromolecular structures (T_21_), the water that is more mobile and trapped in a protein-dense network (T_22_), the loose water in the extra-protein network space (T_23_) (42, 43). In the biscuit system, the three populations were assigned to the three water states of bound, immobilized and free water, respectively.

As shown in Figure 5B, the increasing LSOPC concentration was seen to change both the relaxation durations of the water in biscuit models. The relaxation time of water contained in macromolecules (T_21_) and the loose water in the extra-protein network area (T_23_) were observed to be no significantly difference (*p* < 0.05), whereas, T_22_ exhibited a significant increase with the increasing LSOPC concentration. Thus, it may be concluded that the water mobility in the network of biscuit models to its interior macromolecular structures was increased by LSOPC.

Figures 5C,D presented the water activity and moisture content of the samples at different LSOPC concentrations. After the LSOPC addition, the water activity values ranged between 0.75 ± 0.008 and 0.71 ± 0.005. The rate of the MR likewise rose as the water activity increased, peaking in the range of water activity of 0.65 to 0.75 (44). And the water content ranged between 2.41 ± 0.08% and 3.59% ± 0.31%. The moisture content decreased overall compared to the previous study, which found that “Corn starch biscuits had a moisture content of 6.68 ± 0.06% (45), “even if there was no discernible pattern with LSOPC addition. It could be inferred from the above conclusions that LSOPC could also decrease the formation of AGEs by reducing the Aw of tough biscuits.

### pH properties

**Figure 6:**
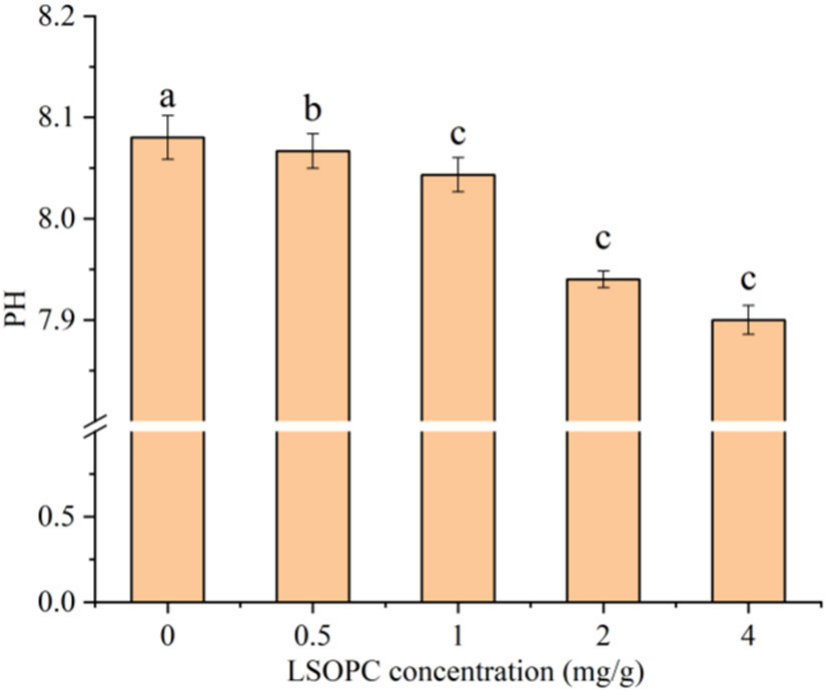
PH of different concentrations. Significant differences (*p* < *0.05*) of data values were indicated by different letters.

In Figure 6, it is depicted how the concentration of LSOPC affected the pH values that changed in biscuit model after baking. Initial pH of biscuit model was higher, and it strongly inhibited the formation of AGEs. In the LSOPC concentration range of 0–4.0 mg/g after heating, higher LSOPC concentration correlated with lower pH. The pH was decreased to 8.08, 8.07, 8.04, 7.94, and 7.90, respectively, which is more intriguing. The MR, which generated organic acids (such as formic acid and acetic acid), is responsible for the pH drop (46). The amino group was reduced, which also likely made the MR system more acidic. It is widely known that the interaction between the amino acid side groups of proteins and lipid oxidation products, which are the primary source of carbonyl compounds, leads to the oxidative cleavage of the carbon backbone of proteins (47). However, in acidic environments, protein tyrosine groups are more quickly oxidized, which could lower the amount of carbonyl compounds produced (48). These compounds underwent further interactions with the side chains of peptides or proteins bring about the formation of AGEs. The aforementioned findings suggested that pH might lessen the production of AGEs by lowering the carbonyl compounds content in biscuits.

### Color properties

**Figure 7:**
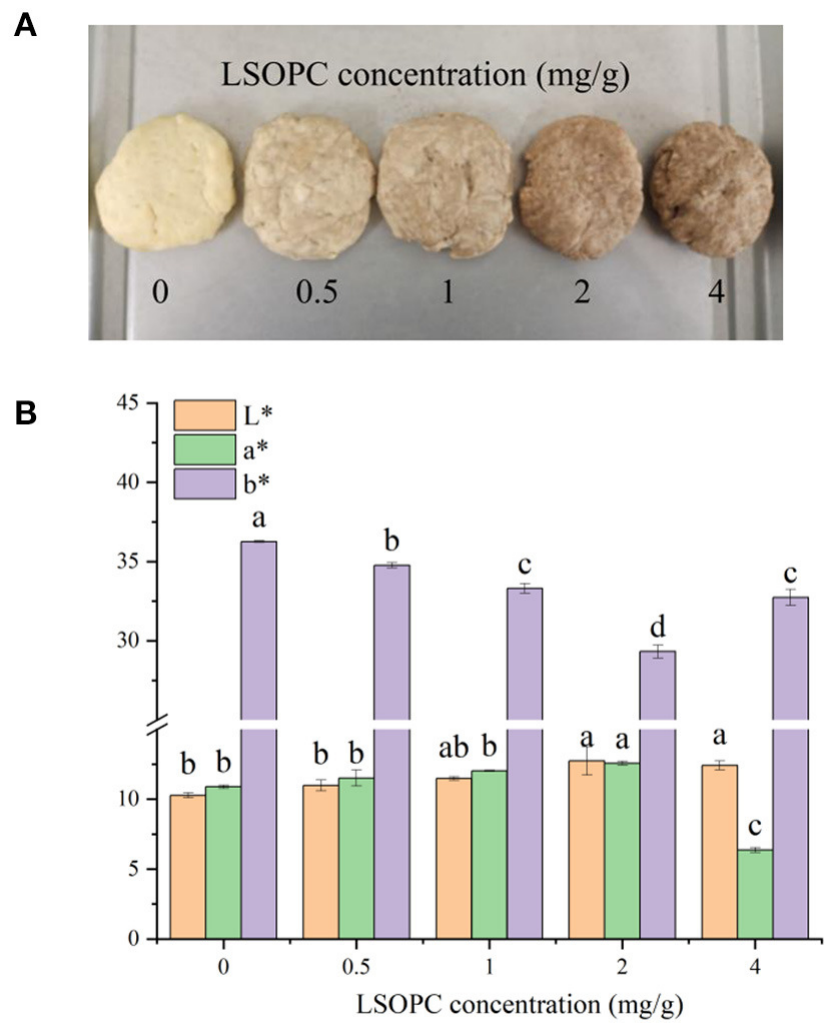
Original images of biscuits **(A)**. Chromatic value of toughed biscuit and the biscuits **(B)**. Significant differences *(p* < 0.05*)* of data values were indicated by different letters. L*: lightness, a*: red/green value, b*: blue/yellow value.

The third stage of the MR, where condensation of carbonyls and amines creates brown-colored high molecular weight molecules known as melanoidins (browning), has been linked to color development. LSOPC was incorporated in biscuit at concentrations of 0.5, 1.0, 2.0, 4.0 mg/g, a noticeable difference was observed on the color in contrast to the control biscuit (0 LSOPC mg/g biscuits) (Figure 7A). In order to accurately analyze the manifest color of the samples, the color difference values of the biscuit models were measured as illustrated in Figure 7B, the parameters L^*^, a^*^ and b^*^ were utilized to quantify the change in biscuit models. The L^*^, a^*^ values of biscuit models were improved with rising LSOPC concentration which below 4 mg/g (*p* < 0.05). It was unequivocal that the higher LSOPC concentration favored the formation of brown color. A substantial chromatic aberration was seen with increasing LSOPC concentration (*p* < 0.05). From a point of view, such color change might owe to the initial color of the LSOPC largely, or to a lesser measure, any colorant formed by the interaction of the LSOPC with the biscuit matrix.

### Texture properties

Another important sensory quality of food products is hardness. Food products typically become harder as a result of moisture loss, plasticizer/moisture redistribution, phase transitions, and protein modification and aggregation. Different LSOPC concentrations were evaluated for textural alterations (Table 1). The hardness of the tough biscuit exhibited irregular differences (*p* < 0.05) when LSOPC concentration increased. The distribution and migration of water in the samples corresponds to the possibility of the redistribution of moisture in biscuits, which might result in the varying hardness.

**Table 1:** Color properties and texture properties of biscuit with different concentrations (0, 0.1, 0.25, and 0.5 mg/mL) of LSOPC.

**Samples**	**ΔE**	**Hardness**
0	–	1,340.94 ± 14.34^b^
0.5	1.66 ± 0.21^c^	3,459.04 ± 31.57^a^
1.0	3.75 ± 0.80^bc^	1,343.11 ± 39.70^b^
2.0	8.34 ± 0.98^a^	1,114.66 ± 50.54^b^
4.0	4.72 ± 0.83^b^	1,411.90 ± 55.23^b^

The data were given as mean ± S.D. (n = 3). Different letters indicated a significant difference (p < 0.05).

### Electronic nose principal component analysis (PCA) in tough biscuits

**Figure 8:**
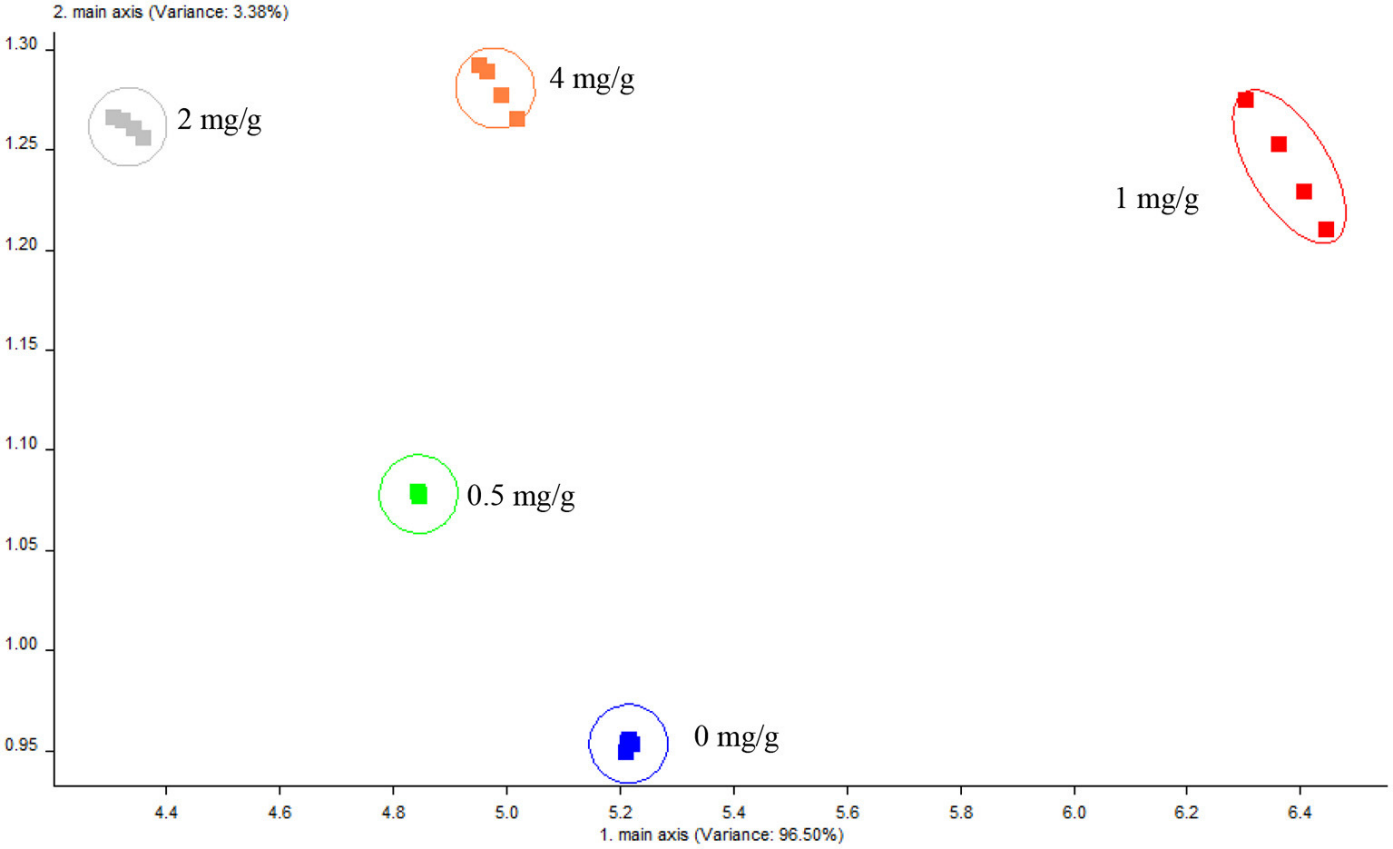
Flavor signals of biscuits.

A type of electronic instrument was electronic nose, which mimicked the human olfactory sense (49, 50). The response values of the sensors were examined using PCA. In Figure 8, each circle delegates a sample, and the space between any two points indicates how different the samples are from one another (51).

The first principal component's contribution rate was 96.50%, and the second principal component was 3.38%. The two principal components' combined contribution rate was 99.88%, meaning that they had successfully captured the majority of the sample's key informational characteristics. Furthermore, the two principal components' cumulative contribution rate was over 99%, indicating that they well captured the key informational properties of the sample, and could be utilized to examine the correlation of volatile components across other samples. PCA imaged of the five LSOPC tough biscuits samples did not overlap each other, indicating that they were different odors and can be distinguished by electronic nose.

Volatile flavor substance Loadings sensor contribution analysis in cooked samples are summarized in Table 2. Despite the fact that every cooked sample had the entire sensor contribution, there were no significant variations (*p* > 0.05) in the ratio of sensor contribution between the five treatments. As a result, the biscuit system could maintain its original flavor with the addition of LSOPC.

**Table 2:** Volatile flavor substance Loadings sensor contribution analysis with different concentrations (0, 0.1, 0.25, and 0.5 mg/mL) of LSOPC.

**LSOPC concentration (mg/g)**		**0**	**0.5**	**1.0**	**2.0**	**4.0**
W1C	Aromatic compounds	1.071 ± 0.004^a^	1.097 ± 0.020^a^	1.129 ± 0.041^a^	1.158 ± 0.062^a^	1.165 ± 0.041^a^
W5S	Very sensitive to nitrogen oxides	2.831 ± 0.256^a^	2.507 ± 0.110^a^	2.477 ± 0.287^a^	2.380 ± 0.166^a^	2.378 ± 0.028^a^
W3C	Ammonia, used as sensor for aromatic compounds	1.052 ± 0.002^a^	1.062 ± 0.008^a^	1.073 ± 0.167^a^	1.083 ± 0.027^a^	1.082 ± 0.014^a^
W6S	Mainly hydrogen, selectively (breath gases)	1.195 ± 0.009^a^	1.193 ± 0.017^a^	1.184 ± 0.009^a^	1.179 ± 0.006^a^	1.187 ± 0.013^a^
W5C	Alkenes, aromatic compounds, less polar compounds	1.043 ± 0.004^a^	1.045 ± 0.004^a^	1.054 ± 0.011^a^	1.057 ± 0.013^a^	1.064 ± 0.023^a^
W1S	Sensitive to methane broad range	1.605 ± 0.064^a^	1.679 ± 0.080^a^	1.772 ± 0.172^a^	1.86 ± 0.235^a^	1.783 ± 0.113^a^
W1W	Reacts on sulfur compounds	2.464 ± 0.236^a^	2.099 ± 0.115^a^	2.402 ± 0.480^a^	2.101 ± 0.178^a^	2.218 ± 0.235^a^
W2S	Detects alcohols, partially aromatic compounds	1.523 ± 0.013^a^	1.576 ± 0.057^a^	1.664 ± 0.117^a^	1.722 ± 0.152^a^	1.693 ± 0.089^a^
W2W	Aromatics compounds, sulfur organic compounds	2.229 ± 0.239^a^	1.949 ± 0.057^a^	1.896 ± 0.238^a^	1.853 ± 0.168^a^	1.833 ± 0.076^a^
W3S	Reacts on high concentrations >100 ppm	1.462 ± 0.005^a^	1.547 ± 0.072^a^	1.598 ± 0.042^a^	1.633 ± 0.053^a^	1.684 ± 0.129^a^

The data were given as mean ± S.D. (n = 3). Different letters indicated a significant difference (p > 0.05).

### Rheological properties

**Figure 9:**
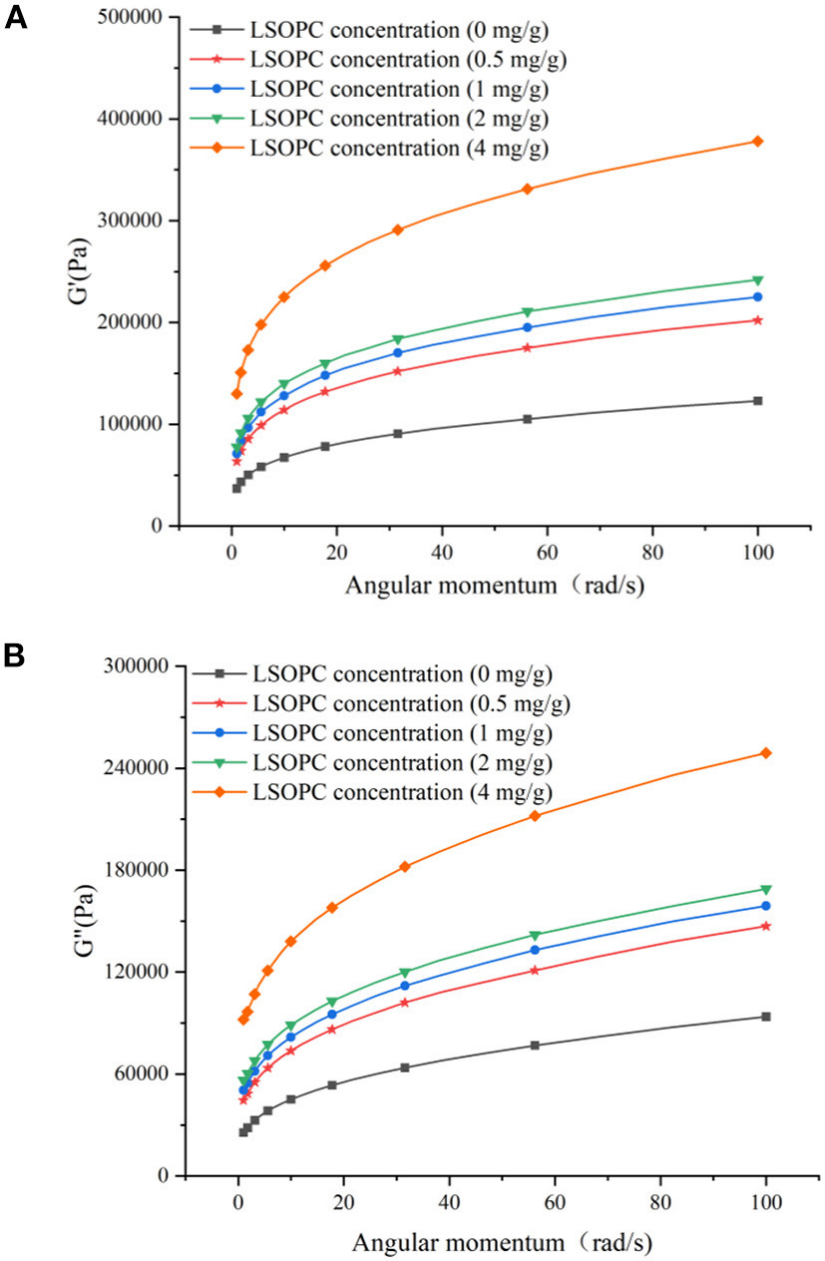
Effect of LSOPC on G′ **(A)** and G″ **(B)**.

The sweep frequency test was used to measure the dynamic rheological properties of dough. The energy recovered per cycle of deformation, which could explain the solid or elastic properties of dough, was calculated using the storage modulus (G'). Additionally, the loss modulus (G') was a measure of the amount of energy lost as heat every cycle of deformation, and revealed the viscous response of dough (52, 53). According to Figure 9, there was a high frequency dependence and an increase in G' and G” with rising LSOPC concentration. In the whole frequency range, G' was >G”, demonstrating the typical rheological characteristics of cross-linking polymers (54, 55). Due to the benzene hydroxyl, LSOPC and gluten generated a significant number of hydrogen bonds, improving the physical crosslinking degree of the gluten network and assisting in the construction of a “grid” structure (Figure 9), which led to an increase in the G' and G” of dough. In conclusion, LSOPC decreased the dough strength, although its active ingredients somewhat improved the toughness and extensibility of gluten dough to some extent. In particular, LSOPC enhanced the lamellar structure of gluten, which benefited on tensile distance. Meanwhile, The “grid” structure was created by the interaction of LSOPC with gluten, which improved the dynamic modulus and strength of dough. The application of LSOPC and its active ingredients in biscuit products will be greatly influenced by these findings.

## Conclusion

According to this study, the development of AGEs in hard biscuits was decreased under LSOPC addition conditions, and the sensory quality was enhanced. Notably, the addition of LSOPC greatly improved the antioxidant activity and AGEs inhibition in biscuits. It was demonstrated that LSOPC prevented AGEs formed by MR in addition to enhancing the functional activity of tough biscuits, which had favorable impacts on health. Additionally, the inclusion of LSOPC decreased moisture content, made tough biscuits harder and more attractive in color, and kept similar flavor. These findings suggested that the sensory quality of hard biscuits containing LSOPC was improved, which would progress the use of LSOPC as a food additive with added value.

## Data availability statement

The raw data supporting the conclusions of this article will be made available by the authors, without undue reservation.

## Author contributions

ZC: conceptualization, methodology, investigation, and writing-original draft. JT: data curation and writing-original draft. JQ: data curation and methodology. NF and CZ: supervision and funding acquisition. QL: data curation. QW: writing-review and editing, supervision, and funding acquisition. All authors contributed to the article and approved the submitted version.

## Funding

This work was financially supported by National Natural Science Foundation of China (Nos. 32001705, 21908048, and 32172185), the Open Project Program of the Beijing Laboratory of Food Quality and Safety, Beijing Technology and Business University (No. FQS-202107), Key Laboratory of Food Nutrition and Functional Food of Hainan Province (No. KF202009), State Key Laboratory of Marine Resource Utilization in South China Sea (Hainan University) (No. MRUKF2021002), and the Collaborative Grant-in-Aid of the HBUT National 111 Center for Cellular Regulation and Molecular Pharmaceutics (No. XBTK-2021003).

## Conflict of interest

The authors declare that the research was conducted in the absence of any commercial or financial relationships that could be construed as a potential conflict of interest.

## Publisher's note

All claims expressed in this article are solely those of the authors and do not necessarily represent those of their affiliated organizations, or those of the publisher, the editors and the reviewers. Any product that may be evaluated in this article, or claim that may be made by its manufacturer, is not guaranteed or endorsed by the publisher.
